# Belowground and Aboveground Responses to Mixed Metal Contamination in Native Central European Trees in Relation to the Species-Specific Autecology

**DOI:** 10.3390/plants15081269

**Published:** 2026-04-21

**Authors:** Madeleine S. Günthardt-Goerg, Rainer Schulin, Patrick Schleppi, Pierre Vollenweider

**Affiliations:** 1 Swiss Federal Institute for Forest, Snow and Landscape Research WSL, Zürcherstrasse 111, 8903 Birmensdorf, Switzerland; patrick.schleppi@wsl.ch (P.S.); pierre.vollenweider@wsl.ch (P.V.); 2 Institute of Terrestrial Ecosystems (ITES), ETH Zürich, 8092 Zürich, Switzerland; rainer.schulin@env.ethz.ch

**Keywords:** apoplast, forest trees, histochemical zinc revelation, leaf economic spectrum, ^15^N, phytostabilisation, plant nutrients, rhizosphere, root tissues, symplast, toxic metals

## Abstract

Using native tree species, the phytostabilisation of toxic metals at former mining and industrial sites can provide ways to prevent metal spread and leaching into the environment and bring the sites back into the economic circuit. In this study, mixed afforestations with young trees from seven Central European species showing contrasted autecology (*Picea abies* (L.) Karst, *Fagus sylvatica* L., *Acer pseudoplatanus* L., *Alnus incana* (L.) Moench, *Populus tremula* L., *Salix viminalis* L. and *Betula pendula* Roth) were exposed during five years to mixed soil contamination (Zn/Cu/Pb/Cd = 1349/317/70/8 mg kg^−1^). The uptake and allocation of the metals in root and shoot tissues, various functional traits and nutrient responses were compared. Despite high metal availability, all tree species showed low metal uptake and similar metal concentrations in their roots. The mobile metals (Zn, Cd) accumulated in the shoot and foliage of early-successional species with acquisitive ecological strategy only, whereas the late-successional species blocked the transfer of all metals from the roots to the aboveground organs. All species showed good tolerance to metal contamination, with large interspecific differences regarding the biomass production and some nutrient concentrations, in apparent relation to the varying species’ ecological strategies and independent of the metal treatment. Zn allocation within fine root tissues could enhance transient spatial and temporal metal immobilisation, especially when associated with protective or defence structures, which also contributed to metal detoxification. Higher transfer of mobile metals to aboveground organs in pioneer tree species was clearly related to their acquisitive ecological strategies, in the context of higher nutrient demand in foliage and lesser defence and protection of vegetative organs. The implications of findings for phytostabilisation applications are discussed.

## 1. Introduction

Soil contamination with toxic metals is a widespread environmental issue, particularly in regions with intensive industrial, mining, or agricultural activities. Metals such as cadmium (Cd), copper (Cu), lead (Pb), and zinc (Zn) pose serious risks to ecosystems and human health through their persistence and bioaccumulation potential [1,2,3]. Various remediation technologies have been developed, among which the phytoremediation methods provide sustainable and cost-effective approaches [4]. The immobilisation of metals in the root zone of spontaneous or planted vegetation reclaiming polluted sites, namely the phytostabilisation, thus aims at reducing metal leaching and dispersal into the environment [5,6]. Using long-lived, high-biomass trees that tolerate elevated metal concentrations while accumulating only small amounts in harvestable tissues may allow the productive and ecological use of contaminated sites for bioenergy, biochar or timber production and landscape restoration, as well as additional benefits for biodiversity, soil fertility and landscape or urban aesthetics [7,8]. Native species often dominate polluted sites such as Cu-Cd-Pb-Zn mining areas [9] and recent reviews have emphasised the ecological and socio-economic advantages of using local plants [10,11] for phytoremediation strategies, primarily phytoextraction and phytostabilisation. However, there is a knowledge gap regarding the effects of species-specific autecology and species mix on the expected phytostabilisation services, which still limits efficient afforestation, subsequent forest management and sound land-use in metal-contaminated areas.

The uptake of metals from soil by plant roots depends on their bioavailability, which in turn is strongly dependent on soil properties, particularly pH and organic matter content. Most metals are more soluble and mobile at low pH, which is typical of many forest soils [12,13]. Only a small fraction of total metal content is available for plant uptake at any time, yet this pool is replenished over time from more stable soil fractions [14]. Root activity further modifies metal availability through pH changes and the release of organic chelators [15]. Mycorrhizal associations, common with forest trees, can improve metal tolerance by binding metals on hyphal walls or secreting metal-chelating compounds [16], while excessive metal exposure may disturb enzymatic processes in the rhizosphere [17]. Differences between tree species have been documented in many studies and experimental systems [18,19,20,21,22]. However, multispecies experiments are less frequent and reports linking metal uptake and allocation from the cellular to the whole-tree level to species ecological strategies are missing. Similarly, comparisons on the relative capacity of native tree species to maintain balanced mineral nutrition in dystrophic conditions because of metal contamination represent another knowledge gap.

The present study builds on previous work using experimental afforestation systems under partly controlled growth conditions [22,23]. Here, we focus on the species-specific ecological strategies and their importance in terms of tolerance and potential phytostabilisation performance. The experimental afforestation system thus included seven temperate tree species, with succession generalists (*Picea abies* (L.) Karst., *Fagus sylvatica* L., *Acer pseudoplatanus* L.) and disturbance specialists (*Alnus incana* (L.) Moench, *Populus tremula* L., *Salix viminalis* L., *Betula pendula* Roth) [24,25,26]. *Picea abies* and *Fagus sylvatica* on the one hand and *Populus tremula* and *Salix viminalis* on the other hand are found at opposite ends of the slow-to-fast leaf economic spectrum (LES) in the case of native Central European tree species [27,28,29]. The experiment was conducted over five years in research plots with either uncontaminated or experimentally contaminated topsoil (Zn–Cu–Pb–Cd = 1349–317–70–8 mg kg^−1^ ≈ 4.5–2.1–0.4–3.8 × legal threshold values [30]) over uncontaminated forest soil, in an experimental garden facility. The experiment aimed to evaluate:

1. How the biomass, morphology, histochemistry and plant nutrition responses to soil contamination with Zn, Cu, Pb, and Cd contrast among tree species with different autecology.
2. Whether these responses are linked to metal allocation patterns within and among plant organs.
3. Which ecological strategies may improve the phytostabilisation performances at forested brown field sites.

## 2. Results

### 2.1. Accumulation and Allocation of Metals

In the metal-contaminated (MC) versus control treatment, the soil concentrations of Zn, Cu, Pb, and Cd were substantially increased in the inserted topsoil as well as subsoil layer, which indicated the displacement of metals from the experimentally contaminated topsoil (Table 1). The inserted subsoil below MC topsoil contained 1.5 (Pb) to 7 times (Zn, Cd) more metal than the inserted control subsoil. During the five-year experiment, the soil pH in the topsoil decreased from 6.55 to 5.3 ± 0.2 and 5.9 ± 0.1 in the control and MC treatment.

**Figure 1:**
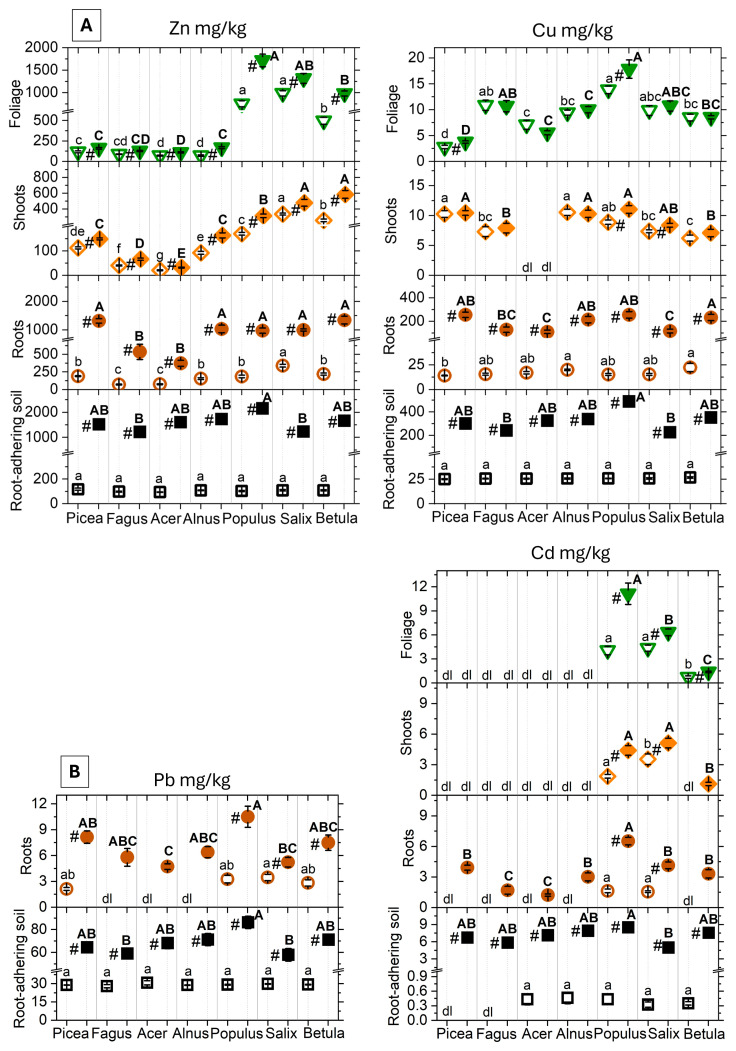
Concentration of metal contaminants ((**A**) Zn, Cu, (**B**) Pb, Cd; mean values ± SE, N = 10 plots) in fine-root-adhering soil (black squares), fine roots (brown circles), shoots orange diamonds), and foliage (green triangles) at the end of the 5-year experimental period. Treatments: control = open symbols, metal contamination = filled symbols. For samples with metal concentrations below the detection limits (dls), dl indications are provided if ≥4 replicates were below threshold. With less than four samples below threshold, half the detection limit was used for the statistical analysis (Cu/Pb/Cd = 1.8/1.5/0.3 mg/kg). In aboveground plant parts, Pb levels remained below detection. Post hoc indication letters and symbols pinpoint the significant differences between the control and MC treatment within species (#), or between species in the control (lowercase letters) and the metal contamination treatment (capital letters; *p* < 0.05, pairwise Tukey’s test). Species are ordered according to their ecological strategies—namely by increasing light requirement (*Picea abies* (L.) Karst, *Fagus sylvatica* L., *Acer pseudoplatanus* L. < *Alnus incana* (L.) Moench, *Populus tremula* L. < *Salix viminalis* L., *Betula Pendula* Roth [24,25,31]) and according to the species niche properties (i.e., *Picea*, *Fagus*, and *Acer* being regarded as succession generalists and the other species as disturbance specialists [26]). Overall significance is summarised in Table 1 and Table 2.

In the topsoil, the metal concentrations in the fine-root-adhering soil fraction versus surrounding material, whatever the treatment, were largely consistent. But metal concentrations were about four times (Zn, Cu) and two times (Pb, Cd) higher in root-adhering soil than those in the bulk subsoil, in the case of the MC treatment (Table 1, Figure 1).

**Table 2:** Main effects of metal contamination (MC), species, and their interactions on element concentrations in root-adhering soil, bioconcentration factors (root/topsoil), and transfer factors (foliage/root) at the end of the 5-year experimental period. Cd transfer factors are shown only for *Populus tremula* L., *Salix viminalis* L. and *Betula pendula* Roth (Cd concentration was below detection limit in the other species). N and C were not determined (nd) in topsoil or root-adhering soil. Pb was below detection limits in all foliage samples. Reported statistics: F-values with significance levels as follows: *** *p* < 0.001, ** *p* < 0.01, * *p* < 0.05, ns = not significant (*p* > 0.05); N = 10 plots.

	Root-Adhering Soil			Bioconcentration Factor			Transfer Factor		
	MC	Species	MC x Species	MC	Species	MC x Species	MC	Species	MC x Species
Zn	3387.06 ***	3.98 *	3.38 *	422.48 ***	77.35 ***	12.16 ***	324.03 ***	126.43 ***	3.86 **
Cu	2285.88 ***	5.00 ***	4.77 ***	11.82 ***	6.50 ***	3.23 **	560.48 ***	45.51 ***	4.45 ***
Pb	775.78 ***	3.53 *	3.10 *	78.68 ***	4.94 ***	2.94 *	-	-	-
Cd	1467.03 ***	3.43 **	ns	4.42 *	35.31 ***	7.09 ***	50.51 ***	14.88 ***	ns
N	nd			nd	-	-	ns	15.7 ***	ns
P	22.38 ***	ns	ns	ns	65.73 ***	ns	62.42 ***	19.62 ***	2.72 *
K	ns	ns	ns	18.75 ***	73.38 ***	ns	6.91 **	29.02 ***	2.33 *
Ca	17.00 ***	ns	ns	ns	99.11 ***	ns	7.50 **	31.70 ***	ns
Mg	6.25 *	ns	ns	5.44 *	65.99 ***	2.86 *	ns	66.62 ***	5.23 ***
S	ns	ns	ns	32.34 ***	206.35 ***	2.30 *	ns	33.53 ***	ns
Fe	79.88 ***	ns	ns	ns	10.46 ***	ns	ns	31.03 ***	ns

Despite the high metal exposure in the MC treatment, the metal uptake in fine roots remained rather low. In the roots from the MC treatment, the Zn, Cu, Pb and Cd concentrations amounted on average to 64, 59, 10 and 47% of those found in the root-adhering soil, respectively. In those from the control trees, they reached 168, 64, and 9% for Zn, Cu, Pb, and for Cd, were near the detection limit (Figure 1). Whatever the element, the metal concentration in the MC versus control treatment was always higher and the mean bioconcentration factors in contaminated plots (BF = root/topsoil) remained lower than 1, with higher values for Zn (0.69) and Cu (0.59), Cd (0.44) than Pb (0.10; Figure S1).

In the MC treatment, the metal concentration in the fine root fractions of the seven tree species showed marked differences: *Picea*, *Salix*, and *Betula* showed the highest Zn (81–87% of the concentration found in the rhizosphere soil) and *Salix* the highest Cd (89%) accumulation. *Acer* and *Fagus* accumulated the least Zn (23 and 44%) and Cd (17 and 30%), whereas Cu and Pb accumulation varied little among species. The variation in the species-specific metal accumulation in fine roots mirrored that observed in the corresponding root-adhering soil fraction (Figure 1).

Metal allocation to the belowground and aboveground organs in the seven tree species proceeded according to an overall trend, with metal accumulation in roots ≫ foliage ≫ shoots (Figure 1, Table 3). The MC treatment significantly increased (1) Zn in all organ compartments, (2) Cu in roots of all species, in shoots of *Populus* and *Salix* and in foliage of *Picea* and *Populus*, (3) Pb in roots of *Picea*, *Populus*, *Salix*, and *Betula* and (4) Cd in roots of all species and in shoots and foliage of *Populus* and *Salix.* Cd and Pb metal contaminants remained below detection limit aboveground in *Acer*, *Alnus*, *Betula* (Pb only), *Fagus* and *Picea*. Overall, *Populus* accumulated the highest metal concentrations in all organs, followed by *Salix* and *Betula*, whereas *Picea*, *Fagus*, *Acer*, and *Alnus* showed low shoot and leaf Zn and Cd accumulation. The aboveground and belowground accumulation of Cu showed less interspecific variation; the lowest values were measured in *Picea* and *Acer* foliage and *Acer* shoots.

**Figure 2:**
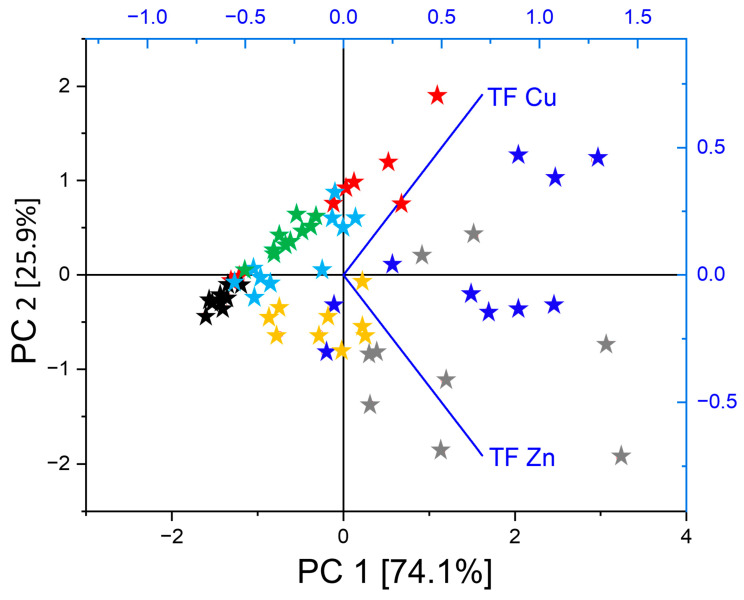
Unconstrained ordination (PCA) of Zn and Cu metal transfer factors (TFs) in tree species with contrasted autecology based on the metal concentrations measured in foliage and root organs in the MC treatment. Ordination of species TF data (*Picea* abies = black, *Fagus* sylvatica = red, *Acer pseudoplatanus* = light blue, *Alnus incana* = green, *Populus tremula* = grey, *Salix viminalis* = dark blue, *Betula pendula* = yellow) within the subspace formed by the first and second principal components (100% of total variance).

The transfer factors (TF = foliage/root) varied significantly with treatment, tree and metal species (Table 2, Figure S1). In *Populus* and Salix, the TFs for Zn and Cd amounted to 2.0 and 1.3 for Zn and 1.7 and 1.6 for Cd in the MC treatment, versus 4.4 and 3.0 for Zn and 2.7 and 2.8 for Cd in the control treatment, respectively. The TFs were generally lower with high metal concentrations in the root systems. The other species either blocked any metal transfer (Cd, Pb) in the MC treatment or showed much lower values with (1) Zn TF in *Betula* (0.8) > *Acer* (0.3), *Fagus* (0.3), *Alnus* (0.2), *Picea* (0.1) and (2) Cu TF *Salix* (0.1), *Populus* (0.1), *Fagus* (0.1), *Acer* (0.06), *Alnus* (0.05), *Betula* (0.04), and *Picea* (0.02). The tree species as a main underlying factor of variation regarding the transfer of metals from the root system to aboveground organs was highlighted by principal component analysis findings (Figure 2). Focusing on the metal TF with values for all tree species (TF Zn, Cu), the seven tree species were primarily separated along PCA 1 (74.1% of total variance). Extreme projection values on this axis corresponded to the *Picea* data group with the overall lowest TFs at the negative axis end and *Salix* and *Populus* groups with the overall highest TFs at the positive axis end. The projection of tested tree species along the PCA 1 then reflected the aforementioned LES gradient [27], considering Central European tree species.

### 2.2. Responses of Functional Traits to Metal Contamination

**Figure 3:**
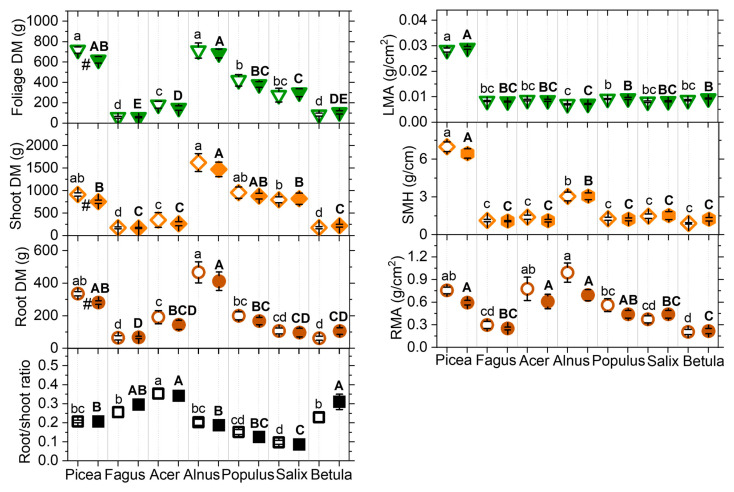
Responses of morphological functional traits (mean values ± SE, N = 10 plots) to metal contamination in relation to tree species autecology by the end of the 5-year metal exposure. Included traits per tree: dry mass (DM) of the roots (brown circles), shoots (orange diamonds), and foliage (green triangles), root dry mass per area (RMA, brown circles), shoot dry mass per tree height (SMH, orange diamonds), leaf dry mass per area (LMA, green triangles), root–shoot ratio (black squares). Treatments: control = open symbols, metal contamination MC = filled symbols. Post hoc indication letters and symbols pinpoint the significant differences between the control and MC treatment within species (#), or between species in the control (lowercase letters) and the metal contamination treatment (capital letters; *p* < 0.05, pairwise Tukey’s test). Species ordination rationale similar to Figure 1. Overall significance is provided in Table 3.

**Figure 4:**
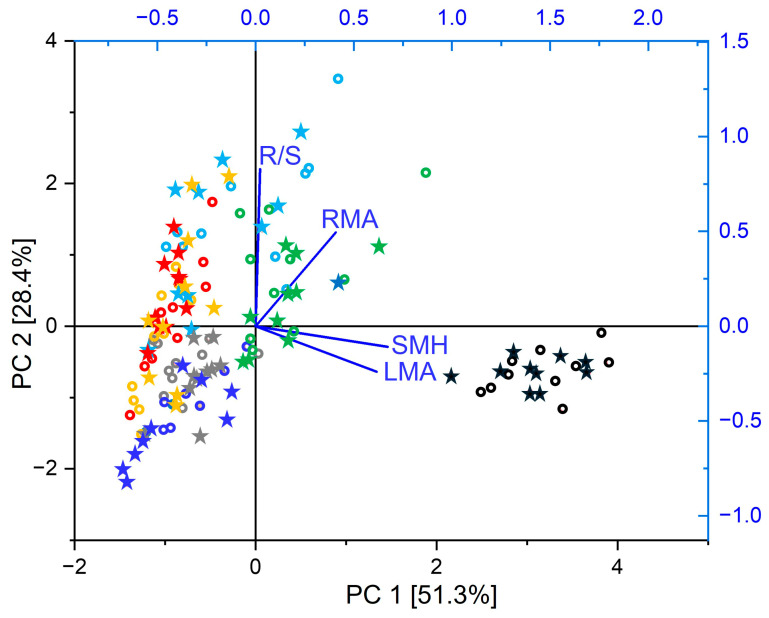
Unconstrained ordination (PCA) of the functional trait ratios based on data measured in foliage, shoots and roots of metal-exposed and control tree species with contrasted autecology. Ordination of root–shoot ratio (R/S), root dry mass per area (RMA), shoot dry mass per height (SMH), and leaf dry mass per area (LMA) data from the control (open circle) and metal contamination (asterisk) treatment (species: *Picea abies* = black, *Fagus sylvatica* = red, *Acer pseudoplatanus* = light blue, *Alnus incana* = green, *Populus tremula* = grey, *Salix viminalis* = dark blue, *Betula pendula* = yellow) within the subspace formed by the first and second principal components (79.7% of total variance).

As observed during the final harvest by the end of the 5-year experiment, the root systems of tested tree species were primarily confined to the topsoil, with deeper roots reaching the inserted subsoil only in the case of *Fagus* and *Acer*. Overall, the seven tree species tolerated well the metal contamination: if the MC treatment reduced the fine-root biomass by 31–47% in *Picea*, *Populus*, and *Salix* (Table 3), it had little effect on other biomass parameters (coarse root, shoot, foliage), specific mass variables (root mass per area, RMA, shoot dry mass per tree height, SMH, leaf dry mass per area, LMA) or biomass allocation (root/shoot ratio; Figure 3, Table 3). Only *Picea* showed significant declines in total root, shoot, and foliage dry mass. Notwithstanding the treatment, the seven tree species showed contrasted growth: *Picea* and *Alnus* produced the highest total biomass, while *Fagus*, *Acer*, and *Betula* achieved the lowest aboveground biomass and *Fagus* and *Betula* the smallest root dry mass per area. *Picea* was characterised by compact growth (high shoot mass per height, SMH) and high leaf mass per area (LMA). Selecting the mass ratio descriptors (independent of tree size), the tree species were separated according to variation in aboveground traits on the PCA 1 axis (51.3% of total variance; Figure 4). The *Picea* data group showed projections on PCA 1 at positive axis end clearly distinct from those of broadleaved tree species. Given the projection of *Salix* and *Populus* at opposite PCA 1 axis extremity, the tree species distribution along this axis reflected well the underlying LES [27], as expected given the species selection rationale (LMA is one of the six spectrum descriptors). It also matched that which was observed in the metal allocation PCA along PCA 1, using TF descriptors (Figure 2). The PCA 2 (Figure 4, 28.4% of total variance) was primarily determined by the variation in biomass allocation and, in a lesser way, by that of the root system morphology in angiosperm trees. *Acer* showed the highest projection values, in possible relation with its root pivot architecture. The MC treatment had no apparent effect on the multivariate ordination of functional traits.

### 2.3. Within-Root Allocation of Zn in Picea and Populus

**Figure 5:**
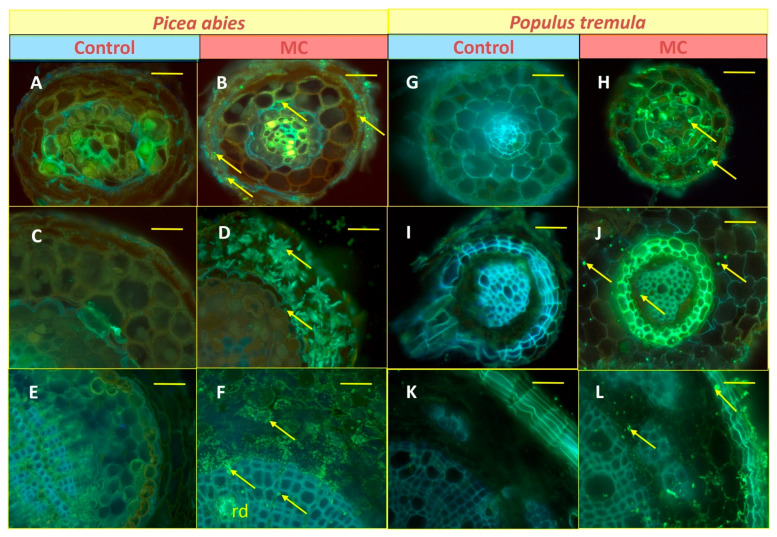
Exemplary microlocalisation of Zn contaminants within root tips (**A**–**D**,**G**–**J**) and fine roots (**E**,**F**,**K**,**L**) of *Picea abies* (**A**–**F**) and *Populus tremula* (**G**–**L**) trees from the metal contamination (**B**,**D**,**F**,**H**,**J**,**L**) versus control (**A**,**C**,**E**,**G**,**I**,**K**) treatment. The Zn contaminants were detected in the form of needle-like greenish fluorescent crystals within the cell symplast (arrows). The Zn levels within roots of control trees remained below the detection limit for histochemical revelation (blue autofluorescence: lignified tissues). Within root tips (**A**–**D**,**G**–**J**), Zn accumulation was found all along the mineral sap absorption and transfer pathway. In fine roots (**E**,**F**,**K**,**L**), it was also observed inside of storage tissues and resin ducts (rd in (**F**)). Root functional zones: (**A**,**B**,**G**,**H**) = absorption zone; (**C**,**D**,**I**,**J**) = conducting zone; (**E**,**F**,**K**,**L**) = perennial root. Technical specifications: 30 µm (root tips) and 60 µm (perennial roots) thick hand-microtomed sections visualised in fluorescence microscopy after histochemical revelation using 8-hydroxyquinoline (excitation band pass filter 340–380 nm, emission long-pass filter 425 nm; [33]). Scale bars: (**A**–**D**,**G**–**J**) = 25 µm; (**E**,**F**,**K**,**L**) = 50 µm.

**Figure 6:**
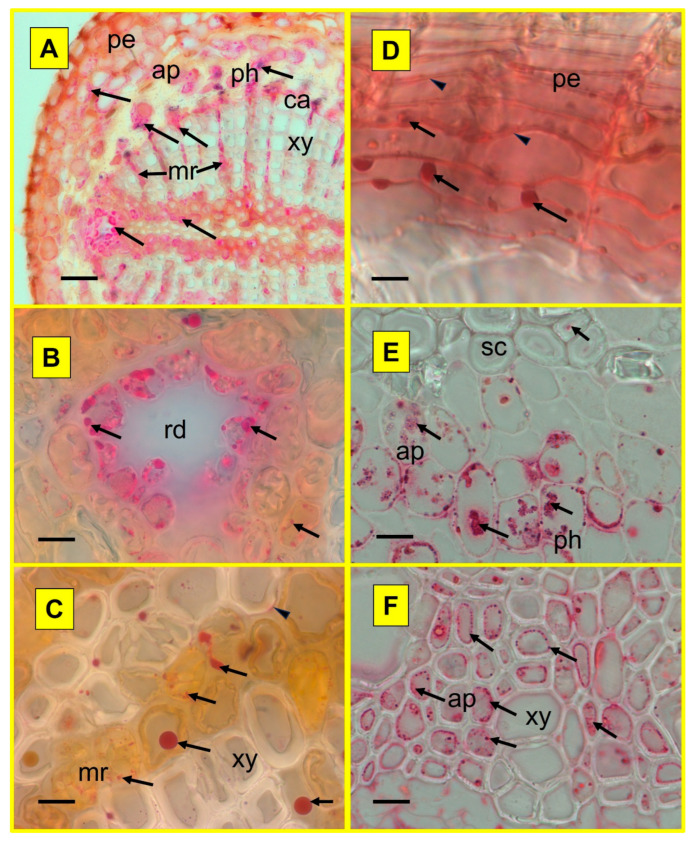
Exemplary allocation of Zn in contaminants at tissue level within perennial fine roots of *Picea abies* (**A**–**C**) and *Populus tremula* (**D**–**F**) trees from the metal contamination treatment. The Zn contaminants were detected in the form of red globoids within cells (arrows) and hues within cell walls (arrowheads). Tissue allocation: storage tissues, namely medullar rays (mr) and axial parenchyma (ap) in xylem (xy, **A**,**C**,**F**) and phloem (ph, **A**,**E**); resin duct (rd, **B**); periderm (pe, **D**). Other tissues: ca = cambium, sc = sclerenchyma. Technical specifications: 60 µm thick hand-microtomed sections visualised in bright field microscopy after histochemical revelation using dithizone (diphenylthiocarbazone; [33]. Scale bar 20 µm (**A**), 12 µm (**B**,**D**) and 2 µm (**C**,**E**,**F**).

**Figure 7:**
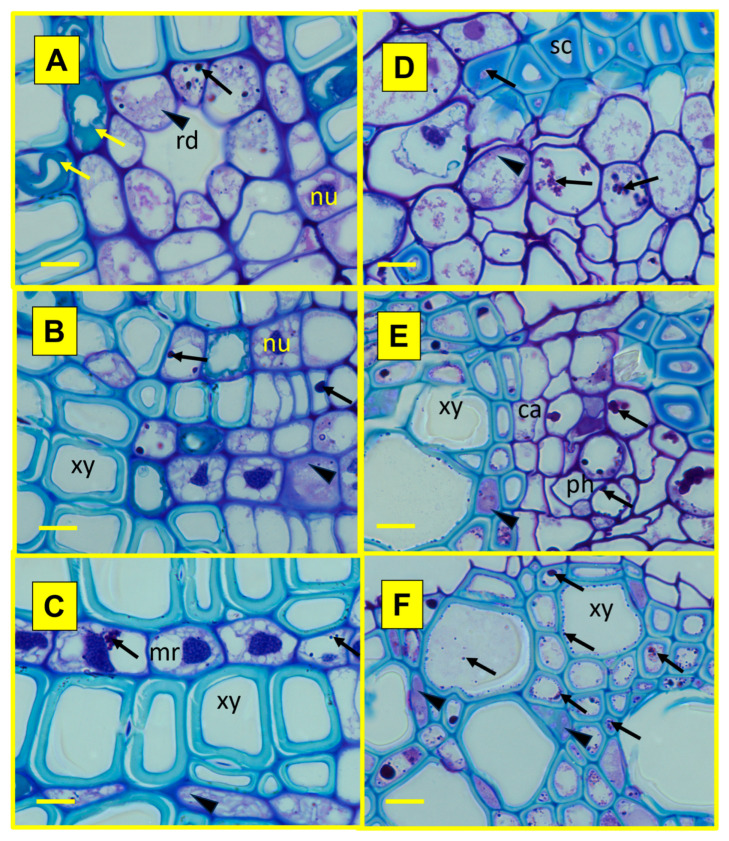
Exemplary cellular allocation of Zn in contaminants within perennial fine roots of *Picea abies* (**A**–**C**) and *Populus tremula* (**D**–**F**) trees from the metal contamination treatment. In the periderm (**A**,**D**) and conducting tissues (**B**,**E**,**C**,**F**), within cells, the Zn contaminants were detected in the form of blue globoids inside of cytoplasm (cy; arrowheads) and vacuoles (va; arrows). Tannins (yellow arrow). Tissue and cell structure: cambium (ca), medullar ray (mr), nucleus (nu), phloem (ph), resin duct (rd), sclerenchyma (sc), xylem (xy). Technical specifications: 2 µm thick semi-thin sections trimmed from Technovit resin-embedded samples stained using acid fuchsine and toluidine blue O. Scale bar: 2 µm (**A**–**F**).

During histochemical analysis, Zn allocation in the fine roots of both selected species with contrasted ecological strategies (*Picea*, *Populus*) could be analysed in the MC and compared to the control treatment. Combining findings from the three histochemical staining techniques, the Zn allocation patterns in different root functional zones and tissues (8-hydroxyquinoline, HQ, Figure 5), within symplast and apoplast (dithizone, DZ, Figure 6) and at a subcellular level (acid fuchsine and toluidine blue O, TBO, Figure 7), could be revealed qualitatively. In the root absorption zone of the two tree species, a Zn signal could be observed overall in all tissues in the MC treatment—and then all along the absorption pathway of mineral sap (Figure 5B,H)—whilst it was rarely found in control samples (Figure 5A,G). In the root conduction zone, mostly similar findings were obtained, with a slight tendency to increased Zn signal frequency (Figure 5D,J versus Figure 5C,I). In the root perennial zone, the Zn signal frequency was clearly increased, and it was also observed in tissues not directly involved in the mineral sap transfer (Figure 5F,L versus Figure 5E,K). A Zn signal was also found in material from control plots at similar tissue locations, with apparent lower frequency. Within vascular tissues, Zn accumulation was especially frequent in axial and radial (medullar rays) storage parenchyma cells within xylem and phloem of both species (Figure 6A,C,E,F). Zn allocation sites in non-conducting tissues included sclerenchyma fibres surrounding phloem (Figure 6E) and the waterproof periderm isolating fine root tissues from surrounding soil (Figure 5K,L and Figure 6D). In spruce, it was also found within defence tissues (resin ducts; Figure 6A,B). Within apoplast, Zn accumulation was generally observed inside the middle lamella of cell walls in the pith of spruce and in the periderm of the two tested species (Figure 6D) or in the lumen of dead xylem cells and resin ducts. Within symplast, Zn accumulated inside cytoplasmic strands—frequently along cell walls, and vacuoles (Figure 6B,E,F and Figure 7). Notwithstanding the species, the globoid shape and β-metachromasy of deposits were indicative of metal chelation by phytic acid ([32]; no signal was detected in control samples because of the lower signal frequency and thinness of semi-thin cuttings).

### 2.4. Changes in Nutrient Concentrations and Allocation in Response to Metal Contamination

**Figure 8:**
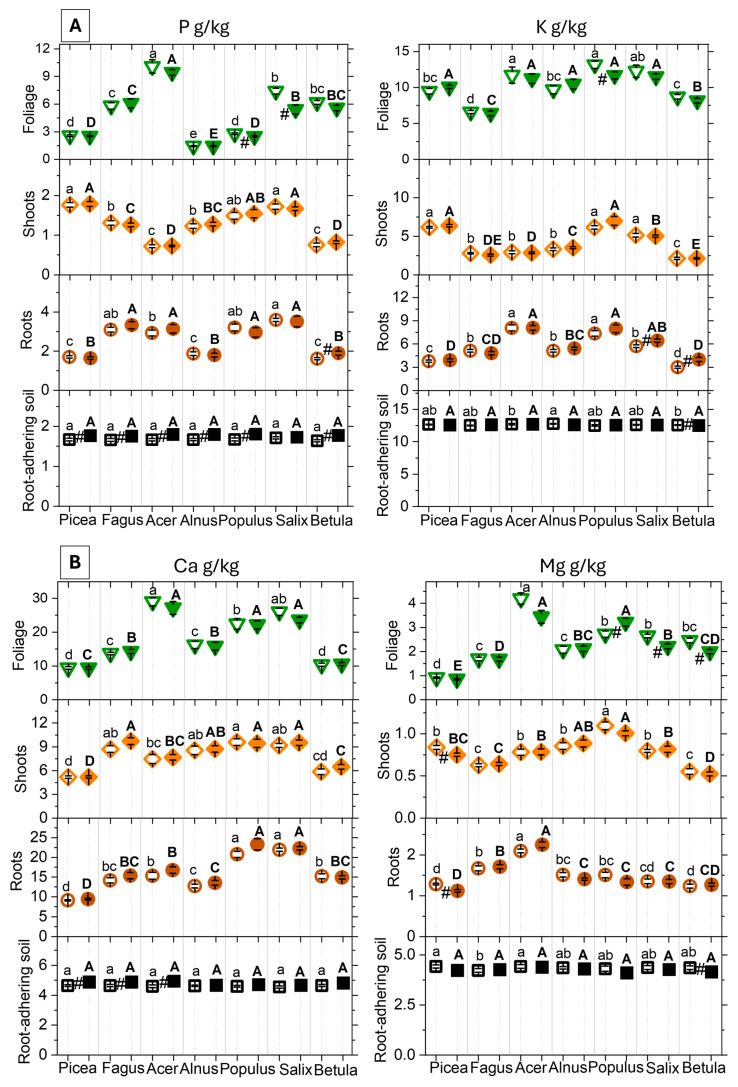

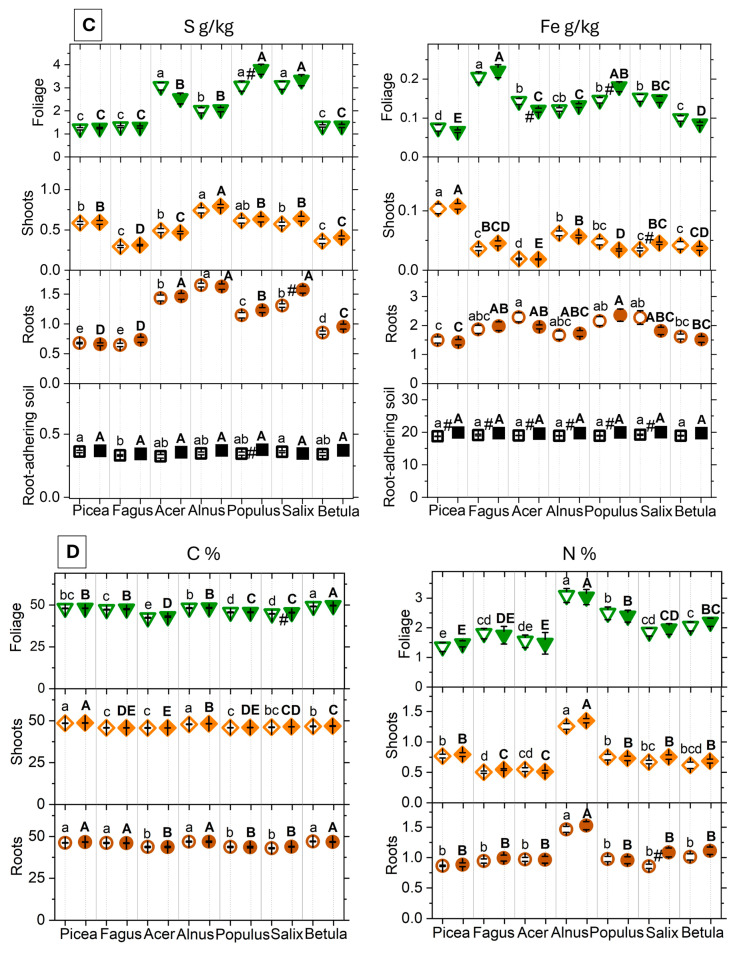

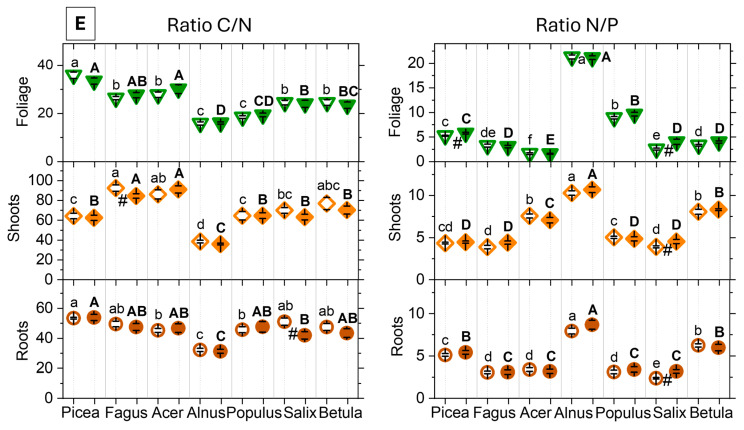
Concentrations of nutrients ((**A**) P, K, (**B**) Ca, Mg, (**C**) S, Fe, (**D**) C, N) and nutrient ratios ((**E**) N/P, C/N) in root-adhering soil (black squares), fine roots (brown circles), shoots orange diamonds), and foliage (green triangles) at the end of the 5-year experimental period. Symbols represent root-adhering soil = black squares, roots = brown circles, shoots = orange diamonds, foliage = green downward triangles, control = open symbols, metal contamination treatment (MC) = filled symbols. Values are shown as mean values ± SE (N = 10 plots). Post hoc indication letters and symbol pinpoint the significant differences between the control and MC treatment within species (#), or between species in the control (lowercase letters) and the metal contamination treatment (capital letters; *p* < 0.05, pairwise Tukey’s test). Overall significance is presented in Table 3.

**Figure 9:**
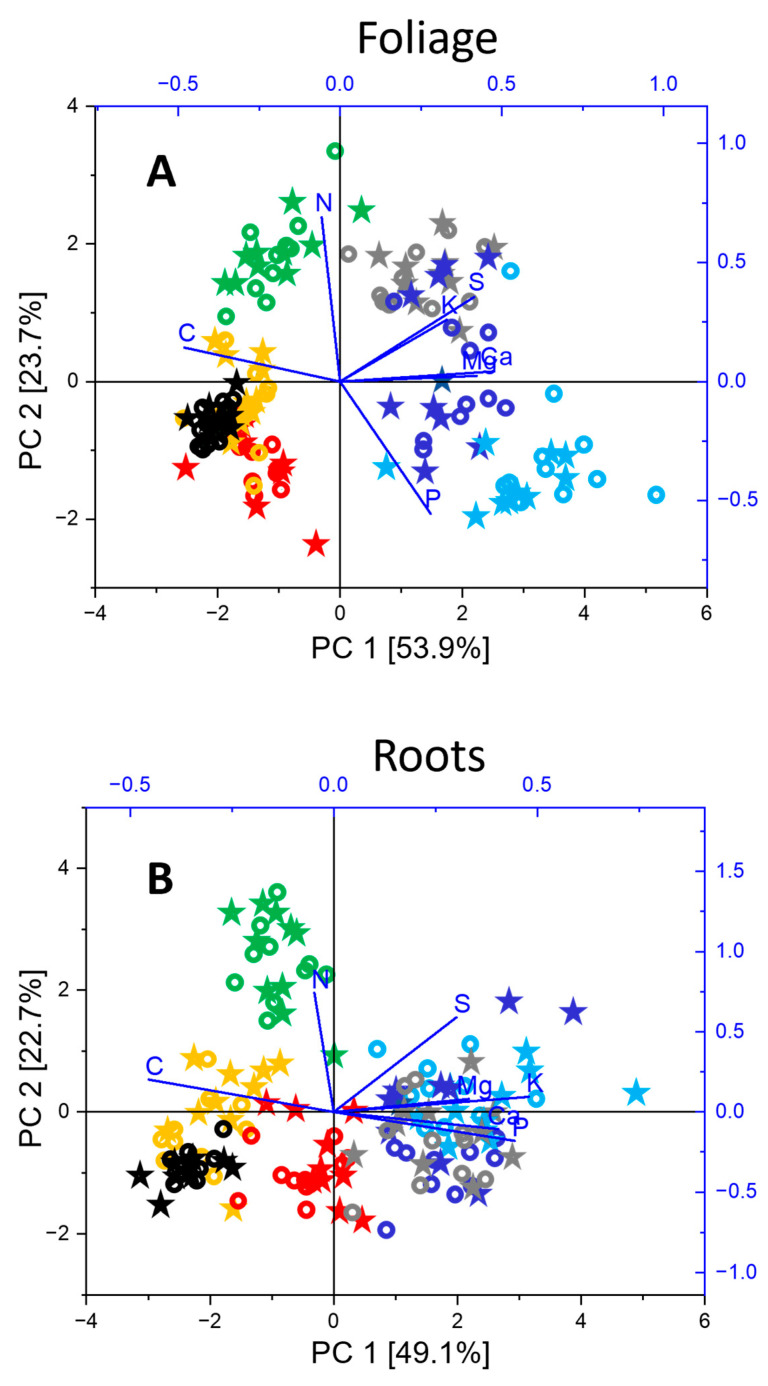
Unconstrained ordination (PCA) of macro-nutrient concentrations (C, N, P, K, Ca, Mg, S) in foliage (**A**) and roots (**B**) of metal-exposed and control tree species with contrasted autecology. Ordination of data from the control (open circle) and metal contamination (asterisk) treatment (species: *Picea abies* = black, *Fagus sylvatica* = red, *Acer pseudoplatanus* = light blue, *Alnus incana* = green, *Populus tremula* = grey, *Salix viminalis* = dark blue, *Betula pendula* = yellow) within the subspace formed by the first and second principal components ((**A**) 77.6% and (**B**) 71.8% of total variance).

The MC treatment induced negligible changes in the nutrient concentrations in bulk soil and caused minor changes in root-adhering soil and plant tissues (Figure 8; Table 1, Table 2 and Table 3). The root-adhering soil showed enrichment in most nutrients (P/K/Ca/Mg/S/Fe) reaching 151/140/185/126/186/127% in the control and 156/82/126/404/204/125% in the MC treatment of values measured in the bulk soil (Table 1; Figure 8). Compared to the root-adhering soil fraction, further enrichment was measured in roots for a few elements (P 150%, Ca 342%, S 323%), whereas the levels of others were reduced (K 45%, Mg 35%, Fe 10%). In the root-adhering soil fraction, the MC treatment slightly raised the concentrations of P and Fe in a general way and those of Ca occasionally, whereas it slightly lowered K and Mg in the root-adhering soil of a few species (Figure 8). In the root organs, the MC treatment (1) lowered the concentration of Mg in *Picea* but (2) increased that of P in *Betula*, K in *Salix* and *Betula* and S in *Salix.* Aboveground, it reduced the shoot concentration of Mg in *Picea* but increased that of Fe in *Salix*. In foliage, the MC treatment (1) lowered the concentration of P in *Populus* and *Salix*, K in *Populus*, Mg in *Salix* and *Betula* and Fe in *Acer* but (2) increased that of Mg, S and Fe in *Populus*. Overall, the concentration of nutrients remained within the sufficiency range limits. The measured macro-nutrient concentrations decreased in the sequence foliage > roots > shoots, while the micronutrient Fe showed a similar pattern to the other metal micronutrients Zn and Cu with the sequence roots > foliage > shoots. Root and shoot nutrients were strongly intercorrelated (P-K, root r = 0.63731 and shoot 0.79761, K-Mg, root r = 0.58732 and shoot 0.6194, P-Ca, root r = 0.57398, *p* < 0.0001). As a general trend, the MC treatment increased the N/P and decreased the C/N ratio (Table 3). *Alnus* generally showed lower C/N and higher N/P than other tree species, whatever the tree organ (Figure 8E). Species with conservative ecological strategy (*Picea* and *Fagus*) tended to have lower amounts of soil nutrients (especially P, K, Ca, Mg, S, N) and higher C/N in foliage than pioneer trees with more acquisitive strategies. Whatever the tree organ, *Alnus* showed higher N concentrations than the other species. Selecting macro-nutrient data measured in foliage (Figure 9A) and roots (Figure 9B), the tree species showed up as the driving factor of variation in the hyperplane formed by the PCA 1 and 2 (Figure 9A,B: 77.6% and 71.8% of total variance), similar to other multivariate findings (Figure 2 and Figure 4). Consistent with the LES rationale [27], the PCA 1 axis in foliage and less clearly root data separated the tree species (*Picea*, *Fagus*) with conservative ecological strategies and higher carbon content from others with more acquisitive strategies and higher soil nutrient concentrations, especially *Populus* and *Salix.* The PCA 2 axis was primarily determined by the N concentration in foliage and roots and driven by the especially high amounts measured in *Alnus*. The MC treatment effects on root and foliage did not affect the multivariate ordination of nutrient data.

**Figure 10:**
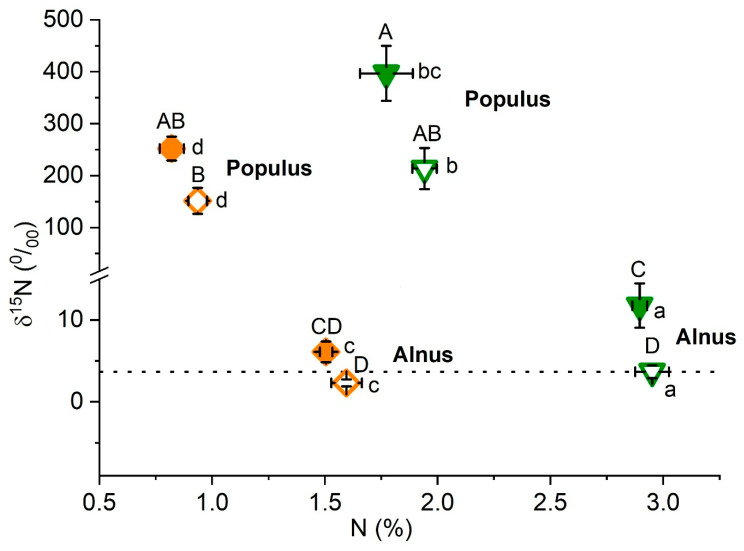
Change in δ^15^N enrichment as a function of N concentration in response to metal contamination in foliage and shoots of *Alnus incana* and *Populus tremula* (mean values ± SE, N = 8 plots). Treatment: control = open symbol, metal contamination = filled symbols; tree organ: foliage = green triangle, shoots = orange diamond. Different capital and lowercase letters indicate significant differences in ^15^N and N concentration (*p* < 0.05, pairwise Tukey’s test). Dotted line = natural ^15^N. Overall significance is shown in Table 1.

^15^N labelling demonstrated that the nitrogen source could strongly differ between the afforestation species, independent of treatment. *Populus* thus showed ^15^N enrichment in both foliage > shoots, with a non-significant tendency to higher uptake under metal contamination (Figure 10). By contrast, the ^15^N levels in *Alnus* remained mostly similar to those in unspiked plots, except a modest increase in foliage under the MC treatment. Notwithstanding the organ and treatment, *Alnus* showed higher N concentrations than *Populus*.

## 3. Discussion

### 3.1. Metal Contamination, Uptake and Allocation in the Afforestation Plots

Contrasting with nutrients, the metal contaminants were not enriched in the root-adhering soil versus bulk topsoil fraction. The tree species factor played a minor role, occasionally significant, regarding metal concentrations in the root-adhering soil fraction. Previous studies have shown that metal solubility and bioavailability in contaminated forest soils is influenced by soil organic matter and pH and is often increased from the bulk to root-adhering soil [34,35,36]; reduced pH under trees, especially conifers, can further increase metal bioavailability [13,37]. However, such effects were not apparent here, in possible relation to experimental settings specificities. Beside Zn and in a lesser way Cd, metal uptake in roots showed few interspecific differences and the observed patterns were in agreement with the literature evidence [38,39]. Hence, the afforestation mixture had low species-specific effects belowground and all species and their combination showed mostly similar metal phytostabilisation potential in the rhizosphere.

Contrasting with trends observed belowground, the species-specific ecological strategy largely determined the allocation patterns of metals observed aboveground in foliage and shoot organs. The mobile metals (Zn, Cd) accumulated in foliage at levels occasionally three times higher than in control trees, but exclusively in pioneer trees and with sometimes TF > 1 (*Populus*, *Salix*, *Betula*). Metals with low mobility were blocked in the rhizospere (Pb) or transferred to levels mostly matching those in uncontaminated material (Cu), independent of tree species. Taking the species separately, our findings largely matched those from model forest ecosystems with higher soil contamination [23], or the observations in trees near smelters [18,40]. Compared to pot studies with increased metal availability and contaminants at higher concentration [22,41], the lesser transfer aboveground of toxic metals (Pb, Cd) observed here depended on the tree autecology; it exclusively occurred (Cd) in pioneer trees. The low BF and TF values were consistent with estimates from forest tree species in mining areas [42,43,44]. The species ordination along PCA 1 according to LES properties, during unconstrained ordination of Zn, Cu TF data, was suggestive of a possible causal role of the acquisitive strategies in pioneer plant species regarding the accumulation of mobile metals aboveground.

### 3.2. Tolerance Indications Under Metal Stress in Tree Species with Contrasted Autecology

After five years, the root systems in the seven tree species were still developing and did not yet show the typical and species-specific root architecture [45]. Those of *Fagus* and *Acer* had reached deeper soil layers and could exploit a larger subsoil volume, which could contribute to the observed lower root metal concentrations. Whilst RMA differences up to three times indicated important interspecific variation, unchanged values under metal contamination were suggestive of unhampered root system development. This may contrast with observations at brownfield sites; our findings then suggest that unfavourable soil physical properties and dystrophic growth conditions in the latter case may impede tree growth at least as much as the toxic metal concentrations [46].

Good metal tolerance in the model afforestation plots, even in the case of *Picea* with minor biomass losses, was indicated by overall non-significant MC treatment effects on dry mass aboveground and in coarse roots, consistent with previous reports [5,7,47]. It was further confirmed by unchanged morphological traits (LMA, SMH) aboveground, in agreement with observations in other studies [23,48]. Indeed, more xeromorphic traits form typical stress reactions in response to various stress factors [49,50]. Enhanced LMA was thus observed in spruce under acute metal stress alone or in combination with elevated ozone concentrations [22]. Uncorrelated LMA and SMH on the one hand and RMA on the other hand reflected the functional independence of aboveground versus belowground organs [29]. The similar species data groups projections on PCA axis 1 in the functional ratios versus TF PCA indicated that the aboveground organs showing lower specific weight could accumulate more mobile metals. This multivariate finding thus confirmed the negative correlations between the accumulation of metals and root/shoot ratio (Cd, Cu Zn), LMA and SMH (Zn, Cu) and RMA (Zn; Table S1). It thus provided a first confirmation that the acquisitive strategies in pioneer trees could enhance metal accumulation aboveground.

### 3.3. Contribution of the Allocation of Metals Within Root Tissues to Metal Tolerance

Given the similar tissue and cell structure in the root absorption and conduction zones of fine root tips whatever the plant species [51], the histochemical patterns observed in *Picea* and *Populus* may be representative of Zn allocation to be found in other studied species. Indeed, metal uptake into roots proceeds through highly conserved structures—notably the endodermis and Casparian strips—filtering solutes and regulating the ion flow [52,53]. Within roots, metals appear (1) to accumulate more in the apoplast than symplast, as shown for Cd by [54,55], (2) to decrease from the outer to inner tissues, and (3) to cross the endodermis barrier by means of membrane transporters before entering the xylem exportation pathway [56,57]. In *Picea* and *Populus*, Zn was observed all along this uptake route. Our results primarily pointed at symplastic allocation, whereas the accumulation of Zn, Cd, Cu and Pb was observed in cortical cell walls of roots in other studies [58,59]. Such pools may contribute to the high metal content measured in the fine roots, but they remain basically exchangeable with the soil solution.

In the root perennial zone of the two investigated tree species, metal allocation similarities and differences were observed, also in relation to each species-specific root structure. Metal allocation to tissues and cells outside the translocation pathway to aboveground organs could occur in the context of nutrient retrieval in storage tissues at the end of the growing season [60]. However, Zn allocation to the impermeable periderm—maybe through the medullar ray, as indicated by the signal in the inner layers and the missing frequency gradient across the periderm—might represent a detoxifying strategy. The pectin-rich middle lamella within the cell walls of, e.g., this latter tissue formed a typical accumulation site inside the apoplast [61,62]. It might improve especially the biological protection in roots, similar to Zn sequestration within resin ducts observed in addition in *Picea*. Such an expedient solution, associating exclusion and defence, was analogous to Ni storage in lactifers and reminiscent of Cd allocation to the collenchyma surrounding leaf veins in some tolerant willows [63,64]. The larger exclusion site availability in spruce material may relate to a higher RMA tendency. High amounts of defence metabolites, notably carbon-rich tannins [22], could also contribute to higher RMA. They were apparently not involved in Zn immobilisation, but they could further slow down the degradation of dead fine roots and then enhance metal stabilisation in the rhizosphere. Within symplast and to the exception of missing signal in plastids, Zn allocation in the root was similar to that observed in leaf cells [65]. In cytoplasm and vacuoles, Zn-stabilisation as phytate complexes—like in leaves—might slow its release in decaying dead roots, compared to free vacuolar ions. Hence, in the root perennial zone—representing a much larger biomass share of the total fine root fraction than the two other zones—the allocation of metals within tissues and rearrangement over time could contribute to further metal immobilisation, even in dead root material. In particular, the species with higher RMA and C-concentration may promote longer-term stabilisation of metals within the soil organic matter.

### 3.4. Nutrient Aspects of Mixed Afforestation Responses to Metal Contamination

Missing dystrophy in the experimental afforestation plots under metal contamination can be ascribed to the selected arable and forest substrates, with balanced soil nutrient concentrations, in contrast with situations at, e.g., mining sites [66]. The higher concentration of macro-nutrients (K, Ca, Mg, P, and S) in the root-adhering versus bulk soil fraction was in agreement with some previous evidence [67] but contrasted with others [68]. Being also observed in uncontaminated conditions, it might primarily relate to larger ion binding capacity enabled by the higher content in soil organic matter in the root-adhering soil fraction. Significant increase by the MC treatment (P, Fe, Ca) might indicate partial restriction of nutrient uptake through competitive inhibition by Zn [21]. However, Zn-induced damage to Mg channels appears unlikely, given the negative treatment effect observed with this latter nutrient, similar to other studies [69]. Overall sufficient and balanced nutrient concentrations in all tree organs confirmed the good tolerance of trees in the model afforestation plots to metal contamination, despite the high concentrations measured in roots or foliage (pioneer trees). This finding contrasted with those in trees under acute metal stress [22], which result in visible symptoms that were inconspicuous here.

Taking the nutrients as descriptors, species data showed species group projections on PCA axis 1 in the PCA using foliage or root data similar to those in the Cu, Zn TF or functional trait PCA. Especially in foliage, species with conservative strategies (*Picea*, *Fagus*) tended to have higher C and lower nutrient content than pioneer trees (especially *Populus* and *Salix*), independent of metal treatment. Beside confirming the importance of the species ecological strategy regarding the response to experimental factors in the model afforestation plots, multivariate nutrient analysis thus provided a mechanistic rationale to explain higher mobile metal transfer in pioneer trees: in agreement with LES [27], pioneer species transferred mobile metals aboveground in the flow of other nutrients to support their higher physiological activity and rapid growth. For similar reasons, they built lightweight organs, with proportionally less carbon and less protective, defensive and detoxifying structures.

The ^15^N labelling treatment provided a complementary mechanistic indication explaining the good tolerance of *Alnus* to metal contamination. Whilst the ^15^N label uptake by *Populus* was consistent with its dependency on the available N sources in the soil, the only slightly raised ^15^N levels in *Alnus* in the MC treatment indicated N-supply primarily through the *Frankia alni* symbiosis, still functional despite the toxic metal environment. In addition to lushy root development [45], this property may be important with a view to successful afforestation establishment in dystrophic soil conditions at brownfield sites. The closely related and also early-successional *Alnus glutinosa* is in addition a specialist of compacted soils, showing larger and quicker root growth compared to other Central European trees. The highest dry mass and RMA observed in *Alnus incana* compared to the other investigated species were also indicative of excellent rooting capacity. Further advantages for phytostabilisation application in *Alnus* included the hampered transfer of mobile metals (Zn, Cd) to aboveground organs, in contrast with other pioneer trees (*Populus*, *Salix*).

## 4. Conclusions

With its mix of early-successional (*Alnus*, *Populus*, *Salix*, *Betula*) and late-successional (*Picea*, *Fagus*) tree species, this study allowed us to compare tree species properties and performance under metal contamination and relate the phytostabilisation potential to the species autecology. Using fertile soils experimentally contaminated, all species were found to be well tolerant to metal contamination, different than what might be found with, e.g., dystrophic and compacted soil conditions at brownfield sites. Compared to the bulk soil, the soil in the rhizosphere showed similar contamination but higher nutrient content. All tree species showed similar metal accumulation belowground. Allocation within root tissues could contribute to transient spatial and temporal metal immobilisation. Defensive structures belowground in species with conservative ecological strategy could further impede mobile metal transfer. Their higher tannin content might also slow the release of contaminants in decaying roots. The transfer of metal to aboveground organs was found to be facilitated by the acquisitive strategies in pioneer trees, in relation to higher nutrient demand to sustain their thriving physiological activity and quick growth. Hence, phytostabilisation applications should prioritise relying on species with conservative strategies and lower nutrient demands. In addition to blocking the metals belowground, their aboveground organs can provide contamination-free products, and their uncontaminated foliage can prevent metal recirculation by leaf shedding. New forest stands, especially at sites with poor soil conditions, may benefit from the remarkable properties found in early-successional *Alnus* trees, including rapid and deep root system development, partly autonomous nutrient acquisition and efficient hindrance of metal transfer aboveground. The conclusions in this study may also apply to other forest species mixes in other industrialised countries and continents, with a similar autecology spectrum.

## 5. Materials and Methods

The experiment was performed using a common garden facility at the Swiss Federal Institute for Forest, Snow and Landscape Research WSL (coordinates: N 47°21′43″/E 8°27′24″, elevation: 550 m), with 20 circular plots, each with a diameter of 2 m, i.e., a surface area of 3.14 m^2^. The upper 0.6 m of the original grassland soil was removed and replaced by a constructed soil consisting of a 0.15 m topsoil layer topping a 0.45 m acidic subsoil layer (pH 4.2 in 0.01 M CaCl_2_), in direct contact with the local subsoil below. The inserted topsoil was removed from a slightly acidic (pH 6.55 in 0.01 M CaCl_2_) loamy arable field soil at Birr, Canton Aargau, with 36–49–15% of sand–silt–clay. The inserted subsoil consisted of a loamy sand taken from a haplic Alisol located in a forest along the river Rhein at Eiken, Canton Aargau, with 87–8–5% of sand–silt–clay. Both soil types were carbonate-free (inorganic carbon < 1 g kg^−1^). Two metal treatments were applied to the topsoil. Ten plots (every second plot) were manually spiked in situ with dust collected from filters of a non-ferrous metal smelter, resulting in mean topsoil HNO_3_-extractable Zn-Cu-Pb-Cd concentrations of 1349–317–70–8 mg kg^−1^, respectively (MC treatment). According to sequential extraction results (1M NH_4_NO_3_, [70]), 85% Zn, 40% Cu, 10% Pb, and 85% Cd were classified as being mobile or easily mobilizable and available for plants [71]. The levels of contamination in this treatment clearly exceeded the Swiss or EU legal trigger values, indicating potentially hazardous soil contamination for arable land (300–150–200–2 mg/kg for Zn-Cu-Pb-Cd, respectively). The concentrations of Cu and Zn were within the range typically found on European industrial brownfields [72], while those of Cd and Pb were within the range considered to be problematic for food production in Europe [73]. The other ten plots with unaltered topsoil metal concentrations of 97–25–30– < 1 mg/kg^−1^ Zn-Cu-Pb-Cd, respectively, served as control treatments. In the local subsoil of both treatments, the initial concentrations were 59–12–20– < 0.1 mg/kg^−1^ Zn-C-Pb-Cd, respectively.

In the year following soil establishment and settling, plant material from seven tree species sourced from local provenances and provided by the WSL nursery were planted in April before bud break. Two trees per species were planted on each plot with full plant-position randomisation: *Alnus incana* (L.) Moench, *Acer pseudoplatanus* L., *Betula pendula* Roth, and *Salix viminalis* L. as bare-rooted, washed 6-month-old cuttings; *Populus tremula* L. as unrooted cuttings; *Fagus sylvatica* L. as 2-year-old seedlings; and *Picea abies* (L.) Karst. as 3-year-old, bare-rooted seedlings, 280 trees in total. All roots were trimmed to 10 cm length. By using these propagation methods, we were able to ensure that the initial weight of the various tree species was similar. An understory layer was planted in each plot but was not assessed here.

^15^N with an isotopic enrichment of 99% was applied as nitrate at a rate of 0.008 mol/m^2^ to 8 plots per treatment (control and MC each) at the beginning of the second season. The remaining two plots in each treatment were left unlabelled to provide baseline measurements for natural ^15^N abundances. Foliage and shoot samples from one *Populus* and one *Alnus* tree per plot were analysed for ^15^N using isotope ratio mass spectrometry at WSL central laboratory at the end of the growing season.

Five growing seasons after planting, the responses to treatments of planted tree species were assessed, measuring growth responses, metal and nutrient concentrations. Therefore, all plants were harvested and separated into foliage, shoot and belowground fractions. Small soil particles still adhering to fine roots after shaking were carefully removed and collected separately. After washing and measuring the projected root system area, the roots were subdivided into fine (<2 mm) and coarse root fractions. The stem height, leaf area and the dry mass of fine roots, coarse roots, shoots and foliage were determined. For comparison with the leaf mass per unit leaf area, the root dry mass per projected root system area was determined, as it better reflects the multidimensionality of root architecture and correlates more strongly with the soil fertility than the specific root length [74,75]. The specific shoot dry mass was estimated using the ratio of shoot dry mass to tree height (SMH).

The concentration of metals and nutrients in the soil layers was determined prior to the plant harvest. The topsoil and subsoil in each plot were sampled, coring three replicates per soil layer which were pooled for analysis. The soil, root-adhering soil and plant organ fractions were dried to a constant weight at 65 °C and homogenised. For chemical element analysis, subsamples were finely grounded using a Retsch MM2000 zirconium oxide-organ bowl ultra-centrifuge mill (Retsch GmbH, Hann, Germany) prior to digestion using a high-pressure microwave digesting system (ultraClave, Milestone, Sorisole, Italy) at 240 °C and 12 MPa. C and N concentration were determined using a gas chromatograph (NC-2500, Carlo Erba-Instruments, Wigan, UK). Contaminants (Cd, Cu, Pb, Zn) and nutrients (Ca, Fe, K, Mg, P and S) were analysed using Coupled Plasma Optical Emission Spectroscopy (ICP-OES, Optima 7300DV, PerkinElmer Inc., Waltham, MA, USA). All analyses were performed at the WSL Central Laboratory according to ISO/IEC 17025:2017 (General requirements for the competence of testing and calibration laboratories, International Organization for Standardization (ISO), Geneva, Switzerland, 2017).

For metals, bioconcentration (BF) and transfer (TF) factors were calculated as ratios between concentrations in the fine roots versus topsoil (BF) and foliage versus fine roots (TF) fractions, respectively.

Based on prior studies of Zn allocation within leaf organs [33,65], we performed histochemical assessments in root material from two species (*Picea abies*, *Populus tremula*) at the opposite ends of the aforementioned LES [27,28,29]. Therefore, healthy attached fine roots (diameter < 2 mm) were sampled in 4 plots per species and treatment before the extraction of belowground organs. They were kept fresh in sealed bags at 4 °C after short washing, waiting for histochemical analysis in the following days, to reduce risks of metal dislodging and leaching. Microscopical preparations were realised selecting three functional root zones, including (1) the absorption zone, with root hairs and active mycorrhiza less than 10 mm away from the root tip, (2) the adjacent conducting zone with emerging lateral roots and inactive mycorrhiza (squeezed root diameter) and (3) the perennial root zone, with secondary tissues and peeling cortex. Hand-microtomed sections were trimmed 30 µm (1, 2) and 60 µm (3) thick and specifically stained for Zn using either 8-hydroxyquinoline (HQ) or diphenylthiocarbazone (dithizone, DZ) [33]. All preparations were immediately observed in fluorescence (HQ, epifluorescence filter combination: excitation band pass filter 340–380 nm, emission long-pass filter 425 nm) or bright field microscopy and imaged using a Leitz DM/RB microscope (Leica Microsystems, Heerbrugg, Switzerland and Wetzlar, Germany; Wild MPS 48/52 micrograph system). Additionally, some perennial root zone segments were fixed in buffered 2.5% glutaraldehyde, embedded in Technovit (7100 embedding resin, Kulzer, Wehrheim, Germany) prior to sectioning (2 μm), staining (metachromatic dye acid fuchsin/toluidine O) and imaging, also in bright field microscopy. With this latter method, the accumulation of Zn in the form of phytic acid chelates was detected as violet globoid deposits showing β-metachromasy [32].

The effects of treatment, species and their interactions were analysed by means of general linear models (GLMs) in SAS (version 9.4, SAS Institute Inc., Cary, NC, USA), using log-transformed data and with significance tested using ANOVA (Type III sum-of-squares) followed by Tukey’s HSD test for pairwise comparison between means. The plot was the statistical unit with 10 replicates, and seven tree species per plot (data used for statistics were mean values from 2 trees of each species per plot, except only one tree for ^15^N). Metal concentrations were reported as “below the detection limit” if ≥4 samples of the 10 replicates were below the respective metal’s detection limit. If fewer than 4 values were below this threshold, half the detection limit (Cu-Pb-Cd = 1.8-1.5-0.3 mg/kg) was used for the statistical analyses [76]. The multivariate responses to experimental factors were further analysed by means of principal component analysis (PCA), based on correlation matrices. All figures and PCA analyses were prepared using untransformed data and the Origin Pro 2024b graphic software (Origin Lab Corporation, Northampton, MA, USA).

## Figures and Tables

**Table 1:** Element concentrations (mg/kg) in bulk subsoil and topsoil from control and metal-contaminated treatments at the end of the 5-year experimental period. Different letters indicate significant differences among soil layers and treatments for each element (*p* < 0.05, Tukey’s test). Values are expressed as mean values ± SE (N = 10 plots), dl = values below the detection limit (Cu/Pb/Cd = 3.6/3/0.6 mg/kg).

	Subsoil		Topsoil	
	Control	Metal Contamination	Control	Metal Contamination
Zn	58.78 ± 2.31 ^a^	401.67 ± 32.99 ^b^	97.43 ± 5.82 ^c^	1348.8 ± 88.04 ^d^
Cu	11.80 ± 0.92 ^a^	82.83 ± 6.91 ^b^	24.68 ± 1.54 ^a^	316.65 ± 20.28 ^c^
Pb	20.05 ± 0.30 ^a^	30.45 ± 1.94 ^a^	30.23 ± 0.88 ^a^	69.58 ± 3.53 ^b^
Cd	dl	3.10 ± 0.41 ^a^	dl	7.70 ± 0.60 ^b^
P	639.18 ± 24.72 ^a^	807.20 ± 32.73 ^ac^	1101.3 ± 14.95 ^b^	1128.0 ± 35.77 ^cb^
K	11,575.0 ± 283.33 ^a^	11,485.0 ± 172.46 ^a^	11,325.0 ± 82.71 ^a^	10,775.0 ± 204.55 ^a^
Ca	3831.5 ± 62.43 ^a^	4144.8 ± 115.01 ^a^	4229.00 ± 62.43 ^a^	4328.0 ± 115.01 ^a^
Mg	2640.0 ± 90.09 ^a^	2730.0 ± 130.45 ^a^	2185.0 ± 113.47 ^a^	2002.5 ± 40.49 ^a^
S	51.60 ± 4.74 ^a^	82.68 ± 13.56 ^a^	167.65 ± 4.74 ^b^	192.10 ± 6.68 ^b^
Fe	18,847.50 ± 288.02 ^a^	18,965.00 ± 385.78 ^a^	20,312.5 ± 160.33 ^a^	19,600.0 ± 227.63 ^a^

**Table 3:** Main effects of metal contamination (MC), species (spec), organs (org, nested within trees), and their interactions on tree morphology, elemental concentrations, and isotope fractions at the end of the 5-year experimental period. Species included: *Picea abies* (L.) *Karst.*, *Fagus sylvatica* L., *Acer pseudoplatanus* L., *Alnus incana* (L.) Moench, *Populus tremula* L., *Salix viminalis* L., and *Betula pendula* Roth (δ^15^N measured only for *Alnus* and *Populus*). Reported statistics: F-values with significance levels as follows: *** *p* < 0.001, ** *p* < 0.01, * *p* < 0.05, ns = not significant (*p* > 0.05); N = 10 plots. Abbreviations: RMA = total root dry mass per area, SMH = shoot mass per hight, LMA = leaf mass per area.

	MC	Species	Organs	MC x spec	MC x Org	Spec x Org	MC x Spec x Org
**Tree morphology**							
Mass	ns	202.45 ***	811.93 ***	ns	ns	27.72 ***	ns
Area	7.62 **	116.59 ***	1954.87 ***	ns	ns	161.31 ***	ns
RMA, SMH, LMA	ns	122.86 ***	3355.46 ***	ns	ns	126.04 ***	ns
Fine root mass	6.66 **	28.66 ***		2.22 *			
Coarse root mass	ns	37.51 ***		ns			
Root/shoot ratio	ns	39.51 ***		ns			
Total biomass	ns	92.02 ***		ns			
**Element concentrations**							
Cd	301.58 ***	93.71 ***	15.03 *	6.63 ***	7.62 *	18.14 *	ns
Cu	792.61 ***	27.28 ***	1038.39 ***	5.90 ***	391.16 ***	13.03 ***	1.83 *
Pb	241.10 ***	4.95 ***	roots only	2.94 **	-	-	-
Zn	1420.47 ***	726.13 ***	201.41 ***	9.5 ***	89.24 ***	28.93 ***	2.96 ***
C	ns	248.23 ***	422.57 ***	ns	ns	104.49 ***	ns
N	ns	167.34 ***	4258.82 ***	2.24 *	ns	46.82 ***	ns
C/N	4.46 *	132.66 ***	3819.95 ***	ns	ns	38.44 ***	ns
P	ns	175.90 ***	1523.17 ***	2.18 *	ns	130.85 ***	ns
N/P	10.83 ***	476.73 ***	118.08 ***	4.51 ***	ns	120.96 ***	ns
K	ns	158.97 ***	1246.38 ***	ns	ns	40.43 ***	ns
Ca	ns	221.05 ***	2091.24 ***	ns	5.46 *	60.23 ***	ns
Mg	10.65 ***	158.78 ***	585.89 ***	ns	ns	24.02 ***	3.19 ***
S	8.19 **	304.91 ***	3024.69 ***	3.28 **	ns	34.92 ***	ns
Fe	ns	27.91 ***	4244.21 ***	ns	ns	29.33 ***	2.56 **
**Isotopes**							
δ ^15^N	33.16 ***	770.24 ***	253.25 *	ns	ns	ns	ns

## Data Availability

The original contributions presented in this study are included in the article/Supplementary Materials. Further inquiries can be directed to the corresponding author.

## Supplementary Materials

The following supporting information can be downloaded at: https://www.mdpi.com/article/10.3390/plants15081269/s1. Figure S1: Main effects of metal contamination (MC) on element concentrations ratios: bioconcentration factors (root/topsoil), and transfer factors (foliage/root) at the end of the 5-year experimental period (mean values ± SE, N = 10 plots). Post hoc indication letters pinpoint the significant differences between the control and MC treatment within species (#), or between species in the control (lowercase letters) and the metal contamination treatment (capital letters; *p* < 0.05, pairwise Tukey’s test). For Cd samples with metal concentrations below the detection limits (dls), dl indications are provided if ≥4 replicates were below threshold. Overall significance is provided in Table 3. Table S1: Pearson Correlations Coefficients of transfer factors (TFs) for metal and nutrient elements with total biomass and biomass ratios. Prob > |r| under H0: Rho = 0, number of observations = 140. Metal contamination treatment (MC), root–shoot ratio (RS), biomass per tree (Biom), leaf dry mass per area (LMA), root mass per area (RMA), shoot mass per tree height (SMH).
