# Supplementary material for: Belowground and Aboveground Responses to Mixed Metal Contamination in Native Central European Trees in Relation to the Species-Specific Autecology
Source: Plants (Basel). 2026 Apr 21;15(8):1269. doi: 10.3390/plants15081269 (PMC13120216; doi:10.3390/plants15081269)
Supplement: Supplementary file 1 [file plants-15-01269-s001.zip › Table S1_Mgg.pdf]

	MC	RS	Biom	LMA	RMA	SMH	TFCd	TFCu	TFZn	TFN	TFC	TFCa	TFFe	TFK	TFMg	TFP	TFS
MC	1	0.0408	-0.0639	0.0086	-0.0062	-0.0211	-0.5168	-0.7545	-0.4269	-0.088	0.0692	-0.1717	0.0531	-0.1689	-0.0398	-0.1194	-0.0428
		0.6334	0.4548	0.9196	0.9424	0.8044	<.0001	<.0001	<.0001	0.3009	0.418	0.0426	0.5333	0.046	0.6403	0.1601	0.6159
RS	0.0408	1	-0.3012	-0.0438	0.206	-0.0398	-0.4602	-0.1886	-0.343	-0.3715	-0.3457	0.188	-0.0206	-0.2169	-0.1533	0.4396	-0.2858
	0.6334		0.0003	0.6103	0.015	0.6423	0.0003	0.0279	<.0001	<.0001	<.0001	0.0267	0.8101	0.0103	0.0716	<.0001	0.0006
Biom	-0.0639	-0.3012	1	0.2211	0.6893	0.5587	0.2736	-0.0434	-0.1468	0.1825	0.1175	0.1004	-0.1714	0.1604	-0.1509	-0.6172	-0.1023
	0.4548	0.0003		0.0092	<.0001	<.0001	0.0395	0.616	0.0858	0.0315	0.1698	0.2396	0.0436	0.0593	0.0761	<.0001	0.231
LMA	0.0086	-0.0438	0.2211	1	0.1891	0.8493	-0.239	-0.2475	-0.2368	-0.2826	0.0677	-0.0376	-0.3561	0.4288	-0.4914	-0.1256	-0.0136
	0.9196	0.6103	0.0092		0.0258	<.0001	0.0734	0.0037	0.0052	0.0007	0.4304	0.66	<.0001	<.0001	<.0001	0.1406	0.8741
RMA	-0.0062	0.206	0.6893	0.1891	1	0.4518	0.0759	-0.1379	-0.305	-0.0658	-0.1861	0.3561	-0.1861	-0.1036	-0.1641	-0.314	-0.1393
	0.9424	0.015	<.0001	0.0258		<.0001	0.5714	0.1081	0.0003	0.4402	0.0283	<.0001	0.0277	0.2233	0.0528	0.0002	0.1008
SMH	-0.0211	-0.0398	0.5587	0.8493	0.4518	1	-0.0179	-0.2671	-0.3566	-0.2611	0.0534	0.024	-0.3042	0.4021	-0.537	-0.322	-0.1783
	0.8044	0.6423	<.0001	<.0001	<.0001		0.8941	0.0016	<.0001	0.0018	0.5322	0.7785	0.0003	<.0001	<.0001	0.0001	0.0351
TFCd	-0.5168	-0.4602	0.2736	-0.239	0.0759	-0.0179	1	0.5508	0.8129	0.2386	-0.4844	0.6214	0.2649	-0.1617	0.1296	-0.3582	0.3816
	<.0001	0.0003	0.0395	0.0734	0.5714	0.8941		<.0001	<.0001	0.0713	0.0001	<.0001	0.0445	0.2253	0.3321	0.0058	0.0031
TFCu	-0.7545	-0.1886	-0.0434	-0.2475	-0.1379	-0.2671	0.5508	1	0.613	0.3322	0.0032	0.0427	0.2033	0.032	0.1822	-0.0198	0.2959
	<.0001	0.0279	0.616	0.0037	0.1081	0.0016	<.0001		<.0001	<.0001	0.9707	0.62	0.0172	0.7102	0.0331	0.8188	0.0004
TFZn	-0.4269	-0.343	-0.1468	-0.2368	-0.305	-0.3566	0.8129	0.613	1	0.4926	0.0811	-0.0636	0.0904	0.0048	0.4155	-0.0546	0.4893
	<.0001	<.0001	0.0858	0.0052	0.0003	<.0001	<.0001	<.0001		<.0001	0.3442	0.4569	0.2897	0.9555	<.0001	0.5234	<.0001
TFN	-0.088	-0.3715	0.1825	-0.2826	-0.0658	-0.2611	0.2386	0.3322	0.4926	1	0.2763	-0.2376	0.0873	-0.0207	0.3874	-0.2902	0.5392
	0.3009	<.0001	0.0315	0.0007	0.4402	0.0018	0.0713	<.0001	<.0001		0.001	0.0047	0.305	0.808	<.0001	0.0005	<.0001
TFC	0.0692	-0.3457	0.1175	0.0677	-0.1861	0.0534	-0.4844	0.0032	0.0811	0.2763	1	-0.6389	-0.2129	0.2428	-0.1182	-0.2583	-0.009
	0.418	<.0001	0.1698	0.4304	0.0283	0.5322	0.0001	0.9707	0.3442	0.001		<.0001	0.0119	0.004	0.1658	0.0021	0.9159
TFCa	-0.1717	0.188	0.1004	-0.0376	0.3561	0.024	0.6214	0.0427	-0.0636	-0.2376	-0.6389	1	-0.0125	-0.3377	0.1416	0.0781	0.059
	0.0426	0.0267	0.2396	0.66	<.0001	0.7785	<.0001	0.62	0.4569	0.0047	<.0001		0.8834	<.0001	0.0951	0.359	0.4887
TFFe	0.0531	-0.0206	-0.1714	-0.3561	-0.1861	-0.3042	0.2649	0.2033	0.0904	0.0873	-0.2129	-0.0125	1	-0.328	0.1074	-0.1422	0.116
	0.5333	0.8101	0.0436	<.0001	0.0277	0.0003	0.0445	0.0172	0.2897	0.305	0.0119	0.8834		<.0001	0.2066	0.0937	0.1722
TFK	-0.1689	-0.2169	0.1604	0.4288	-0.1036	0.4021	-0.1617	0.032	0.0048	-0.0207	0.2428	-0.3377	-0.328	1	-0.1005	0.1752	-0.1027

	0.046	0.0103	0.0593	<.0001	0.2233	<.0001	0.2253	0.7102	0.9555	0.808	0.004	<.0001	<.0001		0.2373	0.0384	0.2273
**TFMg**	-0.0398	-0.1533	-0.1509	-0.4914	-0.1641	-0.537	0.1296	0.1822	0.4155	0.3874	-0.1182	0.1416	0.1074	-0.1005	1	0.206	0.4221
	0.6403	0.0716	0.0761	<.0001	0.0528	<.0001	0.3321	0.0331	<.0001	<.0001	0.1658	0.0951	0.2066	0.2373		0.0146	<.0001
**TFP**	-0.1194	0.4396	-0.6172	-0.1256	-0.314	-0.322	-0.3582	-0.0198	-0.0546	-0.2902	-0.2583	0.0781	-0.1422	0.1752	0.206	1	-0.1161
	0.1601	<.0001	<.0001	0.1406	0.0002	0.0001	0.0058	0.8188	0.5234	0.0005	0.0021	0.359	0.0937	0.0384	0.0146		0.1718
**TFS**	-0.0428	-0.2858	-0.1023	-0.0136	-0.1393	-0.1783	0.3816	0.2959	0.4893	0.5392	-0.009	0.059	0.116	-0.1027	0.4221	-0.1161	1
	0.6159	0.0006	0.231	0.8741	0.1008	0.0351	0.0031	0.0004	<.0001	<.0001	0.9159	0.4887	0.1722	0.2273	<.0001	0.1718	
