# Supplementary figures and images for: Belowground and Aboveground Responses to Mixed Metal Contamination in Native Central European Trees in Relation to the Species-Specific Autecology
Source: Plants (Basel). 2026 Apr 21;15(8):1269. doi: 10.3390/plants15081269 (PMC13120216; doi:10.3390/plants15081269)

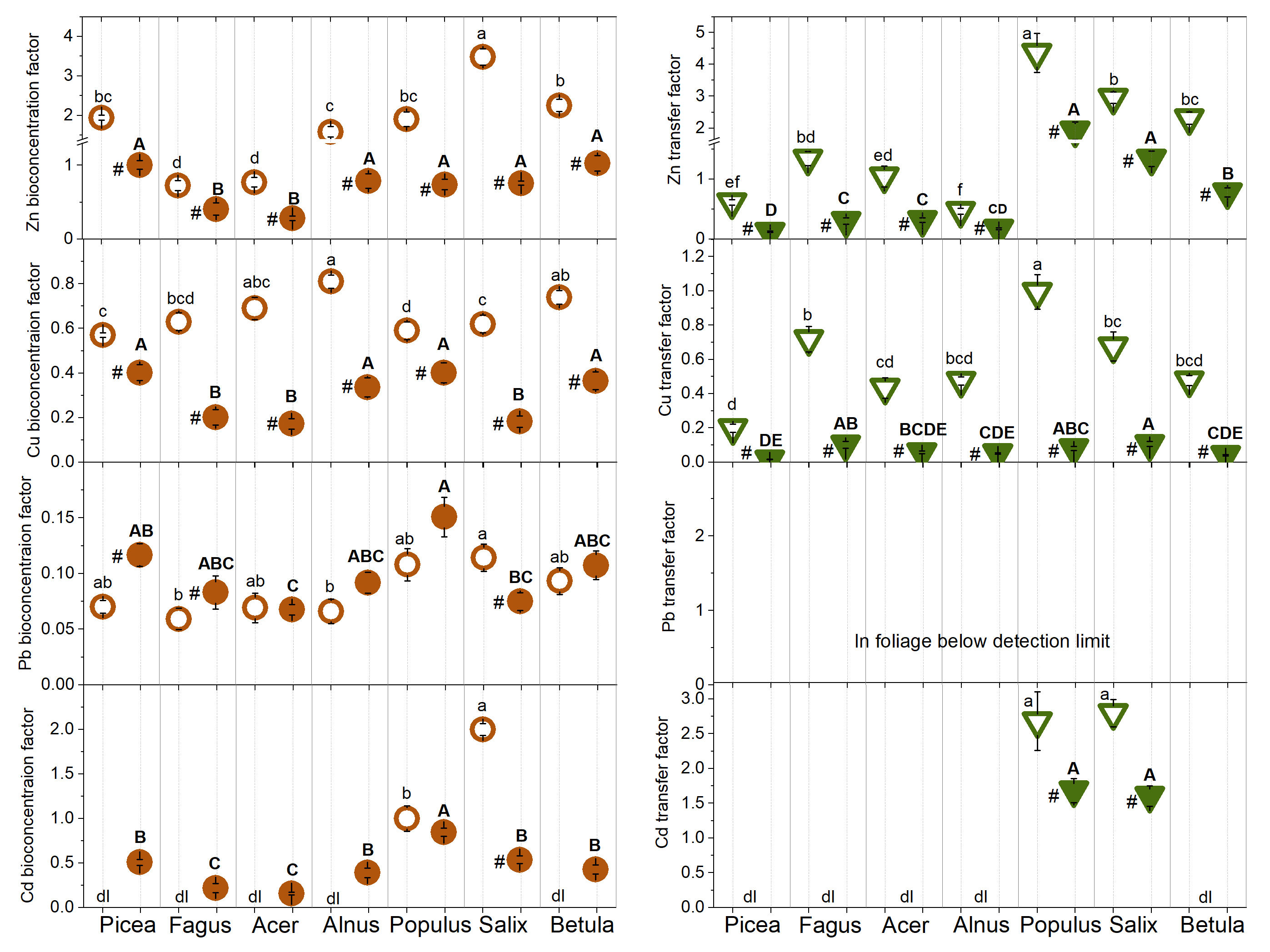

Supplement: Supplementary file 1 [file plants-15-01269-s001.zip › Fig S1_Mgg.tif]
